# Processing of Affective Pictures: A Study Based on Functional Connectivity Network in the Cerebral Cortex

**DOI:** 10.1155/2021/5582666

**Published:** 2021-06-22

**Authors:** Zhongyang He, Kai Yang, Ning Zhuang, Ying Zeng

**Affiliations:** PLA Strategic Support Force Information Engineering University, Zhengzhou 450001, China

## Abstract

Emotion plays an important role in people's life. However, the existing researches do not give a unified conclusion on the brain function network under different emotional states. In this study, pictures from the international affective picture system (IAPS) of different valences were presented to subjects with a fixed frequency blinking frequency to induce stable state visual evoked potential (SSVEP). With the source location method, the cerebral cortex source signal was reconstructed based on EEG signals, and then the difference in SSVEP amplitudes in key brain areas under different emotional states and the difference in brain function network connections among different brain areas were analysed in cortical space. The results of the study show that positive and negative emotions evoked greater activation intensities in the prefrontal, temporal, and parietal lobes compared with those of neutral emotion. The network connections with a significant difference between emotional states mainly appear in the alpha and gamma bands, and the network connections with significant differences between positive emotion and negative emotion mainly exist in the right middle temporal gyrus and the superior frontal gyrus on both sides. In addition, the long-range connections play an important role in the process of emotional processing, especially the connections among frontal gyrus, middle temporal gyrus, and middle occipital gyrus. The results of this study provide a reliable scientific basis for revealing and elucidating the neural mechanism of emotion processing and the selection of brain regions and frequency bands in emotion recognition based on EEG signals.

## 1. Introduction

Emotions are states that combine the physical and mental activities that each of us will produce. Changes in the emotional state will affect our work and life. Positive emotional states are beneficial to improve our happiness in life, and negative emotions can affect our work efficiency and even cause some serious consequences. For example, the negative emotions of fighter pilots may cause plane crashes. Therefore, studying various emotional states and emotional processing processes and understanding the neural basis behind them are of great significance for understanding emotions and effectively identifying and regulating emotions. In recent years, with the development of brain imaging technology, increasing tools provided scientists with a means of directly observing brain activity in emotional states, as well as effective measures for revealing the mechanism of emotion. The current methods used to measure emotional brain activity mainly include fMRI, near-infrared functional imaging, brain magnetism, and EEG. Among them, EEG has become the mainstream method of current emotional brain activity research with its advantages of high time resolution, portability, and low price.

The human brain is one of the most complex systems in nature, and functional differentiation and functional integration are the two organizational principles of human brain function [1]. Scientists have discovered that different areas of the brain dominate different functions, but the brain also needs the coordination of multiple brain areas to complete a very simple task. Emotional activities involve high-level cognitive functions such as memory, cognition, and thinking. The execution of emotional activities is a complex task for the brain. Therefore, the completion of emotional activities requires the interaction and coordination of multiple brain regions, and multiple brain regions will form a complex brain network to process emotions. Therefore, more and more researchers have begun to study emotional activities from the perspective of brain network. Costa et al. used emotional EEG data to construct a phase synchronization brain network. They found that the phase synchronization of the whole brain generally increases in negative emotional states, while phase synchronization occurs between the frontal and occipital lobes in positive emotional states [2]. In 2017, Zhang et al. used granger causality to construct a brain network based on positive and negative emotional EEG signals. They found that the interaction between the prefrontal, parietal, and occipital regions of the brain in the negative emotional state was greater than that in the positive emotional state and the parietal brain area responsible for the human alert mechanism becomes more active in a negative state [3]. In 2018, Wang et al. based on functional magnetic resonance to study the brain functional connectivity of schizophrenia patients during facial emotion cognitive processing, and the results showed that severe schizophrenia patients have amygdala and medial prefrontal cortex in a state of fear and happiness. The functional connectivity between the dorsal anterior cingulate gyrus and the cortex decreased [4]. In 2018, Wyczesany et al. constructed a brain network based on the EEG signals induced by positive and negative emotional face pictures. The results showed that there are strong connections between the frontal lobe, attention network (prefrontal lobe and parietal inner groove), temporal lobe, and occipital lobe in emotional state regions, and the connections of these brain regions are mainly existing in the right hemisphere [5].

In addition, existing studies have shown that frequency band rhythm characteristics are closely related to emotional activity, and many researchers have discovered the characteristics of network connections in different frequency bands. In 2019, Li et al. explored networks with significant differences between positive, neutral, and negative emotions in four frequency bands. They found that network connections mainly exist in the beta and gamma bands, and the network connections in the beta band mainly exist in the forehead, parietal lobe, occipital lobe, and network connections between the temporal lobe and occipital lobe in the gamma band [6]. In 2020, Zheng and Lu studied the network characteristics of various emotional states in five frequency bands. They found that the strong positive network connection in the disgusting emotional state mainly appeared in the gamma frequency band, while the positive network connections in the fear state mainly appeared in the theta frequency band [7]. The above research shows that analysing the characteristics of network connections in different frequency bands plays an important role in revealing the neural mechanism of emotions.

The development of source imaging technology enables the use of EEG recorded by scalp sensors to trace the neural activity in a high-resolution cortical space, thereby realizing real-time imaging of the whole brain neural network [8]. Wheelock found that, under unpredictable threats, dorsal medial PFC is the nerve centre that affects the activities of other brain regions. On the contrary, when the threat is predictable, the dorsolateral PFC is a nerve centre that affects the activities of other brain regions [9]. Ramirez-Mahaluf et al. found that the left dorsolateral prefrontal cortex and the left medial frontal pole are the regulatory regions for the interaction between sadness and cognitive networks [10]. Teckentrup et al. found that the anterior insula cortex can perform different prefrontal network scheduling in the emotional image processing process according to expectations [11]. Keuper et al. studied the cortical response behind emotional word processing and found that, within the P1 time window, the emotional effect peaked in the left middle temporal gyrus [12]. Becker et al. applied source localization techniques to reconstruct the EEG activity on the cortical surface to analyse valence (positive or negative emotions) and they found that source reconstruction can improve the classification results [13]. Cortical spatial brain network connection differences and cortical source response differences are of great significance to better understand the processing of different emotions and improve the accuracy of emotion recognition based on EEG signals. In this work, with the help of stable state visual evoked potential's (SSVEP) good time resolution (time-locked and phase-locked characteristics) and high signal-to-noise ratio, we utilized the source location method to reconstruct the cerebral cortex signal [14]. Then, using source response analysis, SSVEP amplitude analysis, and phase lock value (PLV) functional network connection analysis in cortical space, the neural differences in the processing of different types of emotional pictures are studied from the local and global perspective. This research may provide new evidence for revealing and clarifying the neural mechanism of emotion processing.

## 2. Materials and Methods

### 2.1. The Data Preprocessing

In this study, we used the emotional SSVEP dataset as in our previous work and details about this dataset can be found in our previous study [15]. The dataset consisted of 61-channel EEG signals collected from 20 healthy subjects while they were watching positive, neutral, negative, scrambled pictures flickering at a rate of 10 Hz.

The main preprocessing procedures are as follows:

- Data extraction: the valid data segment is set to −500–2000 ms, in which the length of time before stimulation is 500 ms, and the length of time after stimulation is 2000 ms
- Bad channel average: check whether there is a damaged channel for which EEG data has not been collected, and replace the data of the damaged channel with the data of adjacent channels on average
- Rereference: the data is rereferenced according to the reference electrode standardization technique (REST) proposed by Yao et al. [16]
- Signal filtering: the signal is filtered by 0.1–64 Hz bandpass
- Artifacts removal: set the threshold ±150 uV to eliminate all data segments with amplitude greater than 150 uV
- Baseline correction: baseline correction was performed based on 500 ms before stimulation

In addition, it should be noted that, in the artifact removal step, this chapter does not use the ICA artifact removal method. Instead, referring to the method in the literature, the threshold is set to 150 uV to remove the artifacts with a large amplitude to ensure the original EEG data quality.

### 2.2. Cortical Source Response Analysis

To study the neural differences in the processing of different types of emotional pictures under the conditions of high temporal resolution and high spatial resolution, we use the source localization method to reconstruct the cerebral cortex signals. Then in cortical space, we use source response analysis, SSVEP amplitude analysis, and PLV functional network connection analysis to study the emotional processing mechanism. Source response analysis and SSVEP amplitude analysis can finely explore the activation intensity of local brain areas under different emotional states, while functional network connection difference analysis can show the global interaction of different brain areas. Through the combination of the two analysis methods, the neural mechanisms of different emotional processing will be explored from local and global views.

In the research of this paper, the source reconstruction is carried out with Brainstorm software [17], which is available for free online download under the GNU with general public license (https://neuroimage.usc.edu/brainstorm). Brainstorm software can realize the source imaging of EEG and MEG signals and provides rich time-frequency analysis, connection analysis, and other functions. When using Brainstorm to perform EEG source imaging in this chapter, the anatomical structure of the brain uses the standard brain template ICBM152, the forward model uses the three-layer head model (brain, skull, and scalp) provided by OpenMEEG [18], which is based on boundary elements model (BEM) method [19], and the inverse model uses the minimum norm estimation (MNE) algorithm for source imaging [20].

Source imaging was performed on EEG signals when each subject was viewing positive, neutral, negative, and phase random pictures to achieve cortical signal source reconstruction. Then the source response intensity of multiple subjects was averaged for further analysis; the following are the specific analysis steps.

Suppose the subject *s* is under emotional condition *i*, the EEG signal of the trial *j* is 61 × 1024, 61 is the number of EEG channels, and 1024 is the number of discrete sampling points for each channel (sampling rate 512 Hz; signal length 2 s), where *s* is the subject index, *s* ∈ {1,2,…, *S*}, *S*=20; *i* represents the four emotional conditions, happy, neutral, negative, and random in phase; *j* is the trial index; *j* ∈ {1,2,…, *N*}; and *N* is the number of trials remaining after preprocessing. The number of trails remaining after processing is inconsistent, so no specific value is given, so use *N* instead.

Analysis for each participant is as follows:

(1) Source imaging was performed on **Y**_*s*,*i*,*j*_ to obtain **X**_*s*,*i*,*j*_. In the source imaging operation, the entire cortex is divided into 15,000 dipoles, each trial is mapped to signal **X**_*s*,*i*,*j*_ on the cerebral cortex, and the signal's dimension is 15,000 × 1024.
(2) *N* source imaging results of each trial were averaged to obtain the average source response **A**_*s*,*i*_ of subject *s* under emotional conditions *i*:
  (1) $$\begin{array}{c} A_{s,i}=\frac{1}{N}\sum_{j=1}^{N}X_{s,i,j}. \end{array}$$
(3) *Z*-score standardization processing was performed on the average source response of the subject *s* under the experimental condition *i* to obtain **B**_*s*,*i*_.
(4) Calculate the absolute value |**B**_*s*,*i*_| of **B**_*s*,*i*_.
(5) For each subject *s*, the source response value of neutral emotion is selected as the reference, and the source response values of positive emotion, negative emotion, and phase in random will subtract the value of neutral emotion as follows:
  (2) $$\begin{array}{c} \left|D_{s,i}\right|=\left|B_{s,i}\right|-\left|B_{s,\text{Neutral}}\right|. \end{array}$$
Group analysis is as follows:
(6) The source response differences of all subjects were averaged to get $\bar{D}$:
  (3) $$\begin{array}{c} \bar{D}=\sum_{s=1}^{S}\left|D_{s,i}\right|=\sum_{s=1}^{S}\left(\left|B_{s,i}\right|-\left|B_{s,\text{Neutral}}\right|\right), \end{array}$$

where *s* is the participant index and *S*=20.

$\bar{D}$ reflects the difference in the magnitude of the source response under the experimental condition *i* and the neutral condition. When $\bar{D}>0$, it means that the average source response under emotion is greater than the average source response under neutral emotion; that is, the average source response of emotion is stronger; when $\bar{D}<0$, it means that the magnitude of the average source response under emotion *i* is smaller than that of neutral emotion; that is, the average source response under neutral is stronger.

Neutral emotion was used as a reference, and the differences between the source response values of positive/negative pictures and neutral pictures represent the emotional factor. The differences between the source response values of random phase pictures and neutral pictures represent the influence of semantic factors.

### 2.3. Analysis of SSVEP Amplitude in Cortical Space

Analysing the amplitude of SSVEP in the cortical space can reveal the response strength of different brain areas at the reference frequency 10 Hz and mark the key brain areas involved in emotional processing. We selected 10 Regions of Interest (ROIs) from the cortical space to analyse the SSVEP amplitude. The selection of these ROIs is based on the results of the research on emotion in the literature. Riedel et al. analysed brain activation maps in 1747 experiments, and 5 emotional-related meta-analysis groups were obtained from the entire brain space, that is, primary auditory cortex; insula, anterior cingulate gyrus, and subcortical region; medial prefrontal lobe and posterior cingulate cortex; amygdala and fusiform gyrus [21]. Therefore, we selected 10 ROIs in the cortical PFC, temporal lobe, parietal lobe, and occipital lobe, which are in the cortical PFC, temporal lobe, parietal lobe, and occipital lobe. The emotion-related areas in the cerebral cortex given by Riedel et al. are all included, and each area contains 260 cortical source signals.

Table 1 shows the coordinates of the centre positions of these ROIs in the Montreal Neurological Institute (MNI) space. The centre positions are symmetrical about the left and right hemispheres. In the table, LO, LP, LT, LMF, and LSF represent the middle occipital gyrus, anterior parietal gyrus, middle temporal gyrus, middle frontal gyrus, and superior frontal gyrus of the left hemisphere, and RO, RP, RT, RMF, and RSF indicate the middle occipital gyrus, anterior parietal gyrus, middle temporal gyrus, middle frontal gyrus, and superior frontal gyrus of the right hemisphere, respectively.

The specific analysis steps include the analysis of a single subject and the group analysis of multiple subjects. MOG, APG, MTG, MFG, and SFG represent the middle occipital gyrus, anterior parietal gyrus, middle temporal gyrus, middle frontal gyrus, and superior frontal gyrus, respectively.

The first is the analysis for each subject:

(1) Signal extraction: for subject *s* under emotional condition *i*, extract the cortical signal **X**_*s*,*i*,*r*,*j*_=[**x**_*s*,*i*,*r*,*j*,1_, **x**_*s*,*i*,*r*,*j*,2_,…,*x*_*s*,*i*,*r*,*j*,*m*_,…,**x**_*s*,*i*,*r*,*j*,*M*_]^*T*^ of the trial *j* in the ROI *r*, where *s* is the subject index, *s* ∈ {1,2,…, 20}, *i* represents the four emotional conditions, happy, neutral, negative, and random phase, *r* is the index of the ROI, *r* ∈ {1,2,…, 10}, *j* is the index of the trial, *j* ∈ {1,2,…, *N*}, *N* is the number of trials remaining after preprocessing, and *M* is the total number of signal sources in the ROI, *M*=260. **x**_*s*,*i*,*r*,*j*,*m*_=[*x*_*s*,*i*,*r*,*j*,*m*,1_, *x*_*s*,*i*,*r*,*j*,*m*,2_,…,*x*_*s*,*i*,*r*,*j*,*m*,*L*_]^*T*^ is the length of the first signal in the ROI (sampling rate 512 Hz; signal length 2 s).
(2) Amplitude calculation: Fourier transform is performed on the signal **x**_*s*,*i*,*r*,*j*,*m*_ of each segment, and then the amplitude of the signal *a*_*s*,*i*,*r*,*j*,*m*_ at 10 Hz is calculated.
(3) Averaging of signals in ROI: *M* signals in ROI were averaged to obtain the average amplitude of trial *j* in the ROI *r* of the subject *s* under emotional condition *i*.
  (4) $$\begin{array}{c} b_{s,i,r,j}=\frac{1}{M}\sum_{m=1}^{M}a_{s,i,r,j,m}. \end{array}$$
*M* is the number of signals in the ROI, *M*=260.
(4) Average of trials: the phases of *N* trials were averaged to get the average amplitude of the first ROI of the subject *s* under emotional condition *i*.
  (5) $$\begin{array}{c} c_{s,i,r}=\frac{1}{N}\sum_{j=1}^{N}b_{s,i,r,j}. \end{array}$$
In formula (5), *N* is the number of trials remaining after the subjects are pretreated under the emotional condition *i*.
(5) Normalization: taking the SSVEP amplitude *c*_*s*,Neutral,*r*_ of each ROI under neutral conditions as a reference, the amplitude of each ROI under the emotional condition *i* of the subject *s* is divided by *c*_*s*,Neutral,*r*_:
  (6) $$\begin{array}{c} d_{s,i,r}=\frac{c_{s,i,r}}{c_{s,\text{Neutral},r}}. \end{array}$$
(6) Group average: taking the average of the normalized results *d*_*s*,*i*,*r*_ of all subjects to get the normalized range of the ROI *r* under emotional condition *i*,
  (7) $$\begin{array}{c} {\bar{d}}_{i,r}=\frac{1}{S}\sum_{s=1}^{S}d_{s,i,r}, \end{array}$$

where *s* is the participant index and *S*=20.

In the above process, the normalization is placed before the group averaging, which considers the possible magnitude of the individual differences of each subject. Therefore, the analysis results of each subject are normalized before the group averaging analysis.

### 2.4. Analysis of Cortical Space Function Connection Network

In neuroscience research, the phenomenon of intersignal synchronization is a key feature of information exchange between different regions. Phase synchronization is a commonly used method for constructing undirected networks [22]. The PLV is proposed by Lachaux [23] and PLV calculates the instantaneous phase difference in two signals within a certain narrowband frequency. PLV can quantify the degree of synchronization of two neural signals in a specific frequency band and time zone into a phase-locked state. The larger the PLV value, the stronger the synchronization coupling of the two signals. For two time series *x*(*t*) and *y*(*t*), the calculation process of PLV is as following [23, 24]:

(8) $$\begin{array}{c} \text{PLV}=\left|\frac{1}{L}\sum_{k=0}^{L-1}e^{i\Delta \phi \left(t\right)}\right|,\\ \Delta \phi \left(t\right)=\phi_{x}\left(n\Delta t\right)-\phi_{y}\left(n\Delta t\right), \end{array}$$

where Δ*t* represents the sampling period, *k* represents the number of sampling points, *ϕ*_*x*_(*t*) and *ϕ*_*y*_(*t*) represent the instantaneous phase of the signal *x*(*t*) and *y*(*t*), respectively, and Δ*ϕ*(*t*) represents the phase difference.

According to the division of commonly used frequency bands and rhythms of EEG, the PLV network was calculated in five frequency bands: delta (1–4 Hz), theta (4–8 Hz), alpha (8–12 Hz), beta (12–30 Hz), and gamma (30–64 Hz). The PLV network differences between ROIs under different emotional states are studied by statistical methods. The analysis steps include intrasubject analysis and cross-subject group analysis.

Under the emotional condition *i* of the subject *s*, the cortical space signal of the trial *j* is **X**_*s*,*i*,*j*_, where *s* is the subject index *s* ∈ {1,2,…, *S*}, *S*=20; *i* represents the four emotional conditions, happy, neutral, negative, and random phase; *j* is the index of the trial, *j* ∈ {1,2,…, *N*}, *N* is the number of trials remaining after preprocessing, and the dimension of **X**_*s*,*i*,*j*_ is 15000 × 1024.

Analysis for each subject is as follows:

(1) Signal extraction: for the subject *s* under emotional condition *i*, extract the cortical signal **X**_*s*,*i*,*r*,*j*_=[**x**_*s*,*i*,*r*,*j*,1_, **x**_*s*,*i*,*r*,*j*,2_,…,*x*_*s*,*i*,*r*,*j*,*m*_,…,**x**_*s*,*i*,*r*,*j*,*M*_]^*T*^ of the trial *r* in the ROI *j*, where *s* is the subject index, *s* ∈ {1,2,…, 20}, *i* represents the four emotional conditions, happy, neutral, negative, and random phase, *r* is the index of the ROI, *r* ∈ {1,2,…, 10}, *j* is the index of the trial, *j* ∈ {1,2,…, *N*}, *N* is the number of trials remaining after preprocessing, *M* is the total number of signal sources in the ROI, *M*=260, **x**_*s*,*i*,*r*,*j*,*m*_=[*x*_*s*,*i*,*r*,*j*,*m*,1_, *x*_*s*,*i*,*r*,*j*,*m*,2_, ..., *x*_*s*,*i*,*r*,*j*,*m*,*L*_]^*T*^ is the signal *m* in the ROI, *m* ∈ {1,…, *M*}, and the length of **x**_*s*,*i*,*r*,*j*,*m*_ is *L*=1024.
(2) Principal component analysis: there are two options in Brainstorm “Average” and “PCA.” In order to retain useful component to the greatest extent, we utilize “PCA” (principal component analysis) to extract the largest principal component **d**_*s*,*i*,*r*,*j*_ from **X**_*s*,*i*,*r*,*j*_, as the representation information of ROI [25, 26]. For the subject *s* under emotional condition *i*, the largest principal component **d**_*s*,*i*,*r*,*j*_ of 10 ROI form a matrix under the trial *j* compose matrix is **D**_*s*,*i*,*j*_=[**d**_*s*,*i*,1,*j*_, **d**_*s*,*i*,2,*j*_,…,**d**_*s*,*i*,*r*,*j*_,…,**d**_*s*,*i*,10,*j*_]^*T*^. The dimension of **D**_*s*,*i*,*j*_ is 10 × 1024. Then the PLV connection relationships between the two ROI regions were analysed on **D**_*s*,*i*,*j*_.
(3) Calculating the PLV connection matrices: the PLV connection matrix **P**_*s*,*i*,*j*_ was calculated based on **D**_*s*,*i*,*j*_; **P**_*s*,*i*,*j*_ is a 10*∗*10 network connection matrix. Each value in the network matrix represents the PLV between two ROIs.
(4) Average of trials: the connection matrix **P**_*s*,*i*,*j*_ corresponding to multiple trials were averaged to get the average connection matrix ${\bar{P}}_{s,i}$ of the subject *s* under emotional condition *i*.
  (9) $$\begin{array}{c} {\bar{P}}_{s,i}=\sum_{j=1}^{N}P_{s,i,j}. \end{array}$$
Group analysis of multiple subjects is as follows:
(5) Statistical analysis: paired *t*-test was performed on the PLV connection matrix ${\bar{P}}_{s,i}$ of all subjects under different emotional conditions to analyse whether there are significant differences in the connections between ROIs under different emotional conditions [6, 27].

## 3. Results

### 3.1. Differences of Cortical Source Response

We carried out an analysis of the difference between the source response intensities of the four cases, that is, positive-neutral, negative-neutral, positive-negative, and random-neutral.

#### 3.1.1. Positive-Neutral

**Figure 1:**
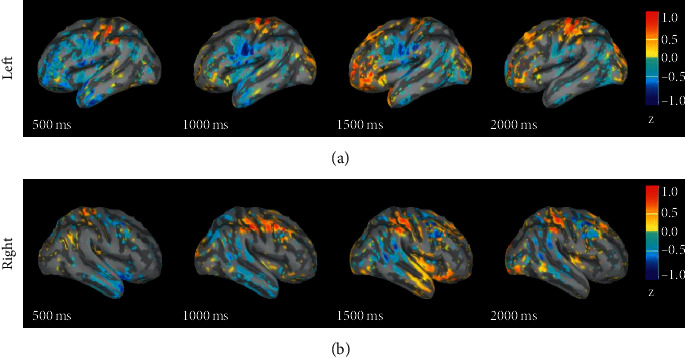
The difference in response intensity between positive and neutral emotional sources.

Figure 1 shows the differences in response intensity between positive and neutral in 0 s to 2 s. The figure shows the differences in amplitude of positive emotion minus that of neutral emotion. As shown by the colour bar, yellow and red represent the amplitude of positive emotion greater than that of neutral emotion. Blue means that the amplitude of positive emotion is less than that of neutral emotion. It can be seen from the figure that, within 0.5 s to 1 s, the activation degree of neutral emotion in the left parietal lobe is greater than that of positive emotion; within 1.5 s to 2 s, the activation degree of positive emotion is greater than that of neutral emotion in the left frontal lobe, right temporal lobe, and double lateral parietal lobes.

#### 3.1.2. Negative-Neutral

**Figure 2:**
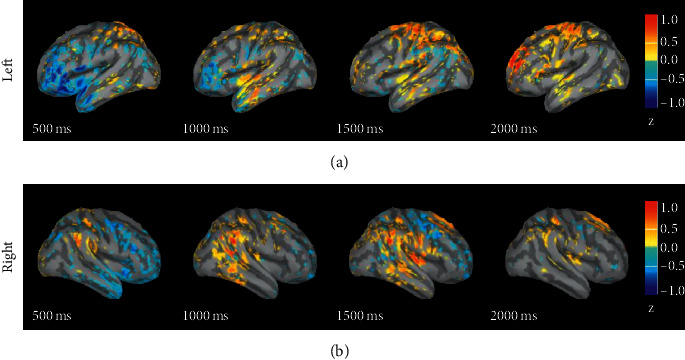
The difference in response intensity between negative and neutral emotional sources.

Figure 2 shows the difference in response intensity between negative emotion and neutral emotion in 0 s to 2 s. The figure shows the difference in amplitude of negative emotion minus that of neutral emotion. It can be seen from the figure that, at 0.5 s, the activation degree of neutral emotion in the left and right prefrontal lobes is greater than that of negative emotion; from 1 s, the activation degree of negative emotion gradually increases in the left hemisphere temporal lobe (1 s), parietal lobe (1.5 s), and the prefrontal lobe (2 s); the intensity is greater than that of neutral emotion; from 1 s, the response of negative emotion gradually increases in the right hemisphere, and the intensity of negative emotion in temporal lobe response is greater than that of neutral emotion.

#### 3.1.3. Negative-Positive

**Figure 3:**
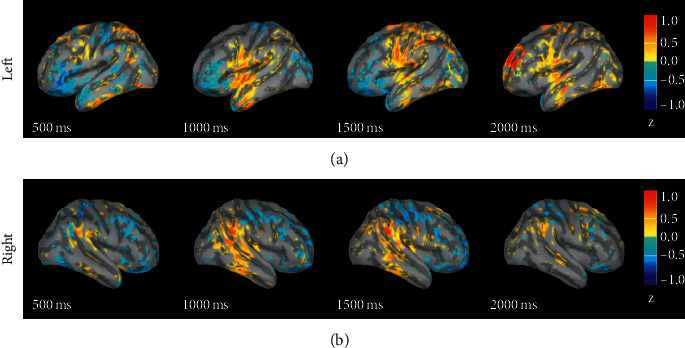
The difference in response intensity between negative and positive emotional sources.

Figure 3 shows the difference in response intensity between negative emotion and positive emotion in 0 s to 2 s. The figure shows the difference in magnitude of the negative emotion minus that of positive emotion. It can be seen from the figure that, from 0.5 s to 1 s, the degree of activation of the negative emotion in the frontal lobe on both sides is lower than that of the positive emotion; starting from 1 s, the intensities of negative emotion in the temporal lobe (1 s), parietal lobe (1.5 s), and prefrontal lobe (2 s) are greater than that of the neutral emotion; from 1 s, the intensity of negative emotion in the temporal lobe of right hemisphere enhanced.

#### 3.1.4. Random-Neutral

**Figure 4:**
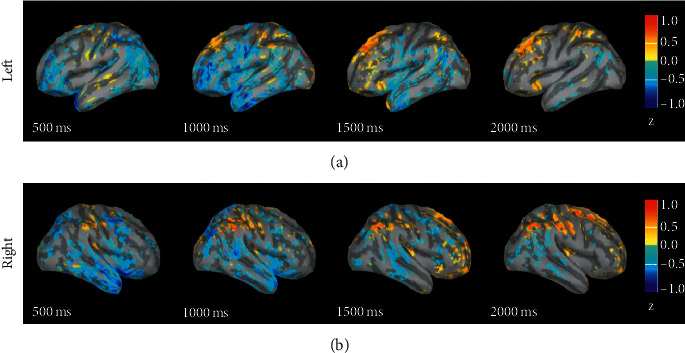
The difference in response intensity between random and neutral emotional sources.

Figure 4 shows the difference in response intensity between phase random picture evoked emotion and neutral emotion in 0 s to 2 s. The figure shows the difference in magnitude of *t* random phase picture evoked emotion minus that of neutral emotion. It can be seen from the figure that, from 0.5 s to 1 s, the degree of response intensities of neutral emotion in the occipital, prefrontal, and temporal lobes on the left and right sides is greater than that of random phase picture evoked emotion; starting from 1.5 s, the response intensities of the parietal lobe in left and right hemispheres gradually enhanced and are greater than those of neutral emotion.

### 3.2. Difference in SSVEP Amplitude

Taking SSVEP amplitude of neutral emotion as a reference, the amplitudes of each ROI under the positive emotion, negative emotion, and phase random picture evoked emotion were divided by the amplitude of each ROI area under the neutral condition, respectively.  Table 2 shows the normalized amplitude of the 10 ROI regions under the positive emotion, negative emotion, and phase random picture evoked emotion on the cortex. It can be seen from the table that (1) the amplitude of positive emotion on the left frontal lobe is higher than that on the right, while the amplitude of negative emotion on the right frontal lobe is higher than that of the left frontal lobe, (2) negative emotion has the largest amplitude in the occipital, temporal, and middle frontal lobes on both sides, and the activation intensity of the ventral pathway is the largest, and (3) except for the anterior central gyrus of the right parietal lobe, the SSVEP amplitudes of phase random picture evoked emotion in the other brain areas are all smaller than those of neutral emotions.

### 3.3. Difference in Functional Network Connection

#### 3.3.1. Positive-Neutral

**Figure 5:**
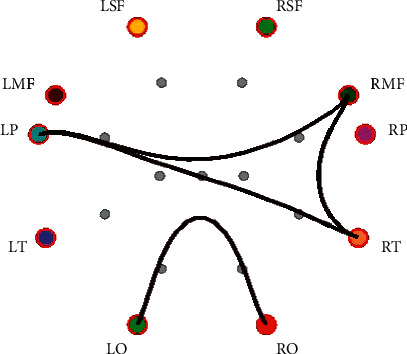
PLV connection with significant difference between positive emotion and neutral emotion in the gamma band (the black lines indicate that the PLV connection values of positive emotion are smaller than those of neutral emotion).

Table 3 shows the PLV connections with significant differences between positive emotion and neutral emotion. There are more differential connections in the alpha and gamma bands. The PLV connections show that key brain areas are RP, RT, RMF, and LMF.  Figure 5 shows the PLV connections with a significant difference (*P* < 0.05) between positive emotion and neutral emotion in the gamma band, and the PLV connection values under positive emotion are smaller than those of neutral emotion.

#### 3.3.2. Negative-Neutral

**Figure 6:**
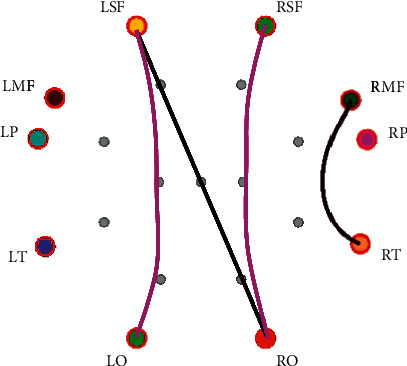
PLV connection with significant difference between negative emotion and neutral emotion in the gamma band (the black lines indicate that the PLV connection values of negative emotion are smaller than those of neutral emotion, and the red line indicates that the PLV connection values of negative emotion are greater than those of neutral emotion).

Table 4 shows the PLV connections with a significant difference between negative emotion and neutral emotion. The PLV connections with a significant difference mainly exist in alpha and gamma bands. The PLV connections show that the key brain areas are RMF, LSF, RSF, and RO.  Figure 6 shows the PLV connections with a significant difference (*P* < 0.05) between negative and neutral emotions in the gamma band.

#### 3.3.3. Negative-Positive

**Figure 7:**
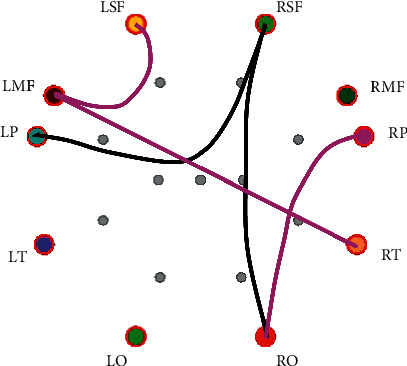
PLV connections with significant difference between negative emotion and positive emotion in the alpha band (the black lines indicate that the PLV connection values of negative emotion are smaller than those of positive emotion, and the red lines indicate that the PLV connection values of negative emotion are greater than those of positive emotion).

Table 5 shows that PLV connections with significant differences (*P* < 0.05) between positive emotion and negative emotion PLV connections with significant differences between positive and negative emotions mainly exist in the alpha, beta, and gamma bands, and there are more connections with significant differences in the alpha band. The alpha (8–12 Hz) wave represents the state of relaxation. With the increase of mental load, the energy of the alpha band will decrease, which is obvious in the temporal lobe and occipital lobe, and there will be energy changes in the frontal lobe under complex tasks. The PLV connections show that the key brain areas are RO, LMF, and RSF. PLV connections with a significant difference between negative emotion and positive emotion in the alpha band are shown in Figure 7.

#### 3.3.4. Random-Neutral

**Figure 8:**
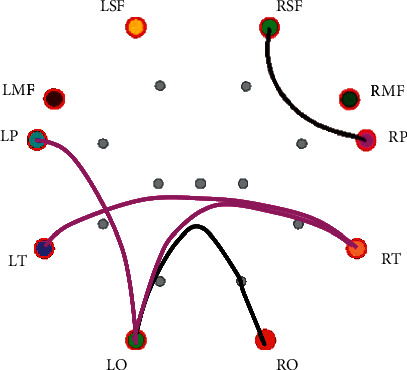
PLV connections with significant difference between phase random and neutral emotion in the delta band (the black lines indicate that the PLV connection values of the random phase emotion are smaller than those of neutral emotion, and the red lines indicate that the PLV connection values of the random phase are greater than those of neutral emotion).

Table 6 shows the PLV connections with a significant difference (*P* < 0.05) between phase random and neutral emotion in each frequency band. The PLV connections with a significant difference mainly exist in delta and gamma frequency bands. The key brain areas are LO, RT, RSF, RT, and LT.  Figure 8 shows PLV connections with a significant difference between phase random and neutral emotion in the delta band.

Based on the EEG source location method, this study explores the dynamic response of the cerebral cortex, the amplitude difference in different emotions at 10 Hz frequency, and the emotional-related brain functional network connection patterns in the process of affect pictures. The neural mechanism of emotion was analysed from the response in local brain regions and differences in network connections between multiple brain regions.

## 4. Discussion

### 4.1. The Difference in the Source Response of Different Emotions

Through the analysis of the difference in the source response of different emotions, we found that the response intensity of neutral emotion is greater than that of positive and negative emotions within 1 s after the stimulus was displayed. After 1 s, the activation intensities of positive and negative emotions are greater than those of neutral emotion in the temporal lobe, parietal lobe, and prefrontal brain areas. A comparison of the source responses of positive and negative emotions shows that the response intensities of positive emotion in the frontal lobes on both sides are greater than those of negative emotion. After 1 s, the response intensities of negative emotion are greater than those of positive emotion in the temporal lobe, parietal lobe, and prefrontal lobe. It can be found that the difference in how the brain processes different emotional stimuli is mainly in the prefrontal brain area. According to previous studies, the prefrontal lobe is a brain area related to high-level cognitive functions. Many studies have also found that the prefrontal lobe plays an important role in emotional processing. Zhuang et al. found that the channels that play an important role in emotion recognition mainly come from the prefrontal, temporal, and occipital brain regions [28]. Lin et al. studied the behavioural and neurological effects of members within and outside the group on individual emotional processing. They used fMRI to observe the ventral striatum and peritoneal prefrontal cortex, and the prefrontal cortex of the rucksack, the medial prefrontal cortex, the supratemporal posterior sulcus, and other brain regions are activated in relation to rewards and positive valence [29]. Perry et al. studied the impact of prefrontal cortex damage on emotional understanding. They found that the accuracy and response time of emotional understanding of patients with prefrontal cortex damage are worse than those of normal people [30]. Their research has once again confirmed that the prefrontal cortex plays an important role in understanding the emotion.

### 4.2. The Analysis of Functional Network Connection

In the functional network connection analysis, we found that network connections with significant differences between different emotional states mainly exist in the alpha and gamma frequency bands. The alpha band is related to the relaxed state, and with the cognitive load of the brain increasing in the emotional state, the alpha band will have a corresponding energy change. Arndt et al. used EEG signals to evaluate the effect of the quality of the speech-to-text system on users' emotion. They found that when the subjects faced lower quality synthetic text, the neuron activity in the alpha band of the left frontal lobe increased [31]. However, Schubring et al. found that when subjects dealt with high arousal emotional stimuli, the energy in the alpha band decreased [32]. In short, energy changes in the alpha band are closely related to emotional stimulation processing. Many previous studies have pointed out that high-frequency EEG plays an important role in high-level cognitive activities such as memory, decision-making, and emotion. Li et al. studied the differences in network connections between emotions in different frequency bands, and they found that there are most network connections with a significant difference in the gamma band [6]. Matsumoto found that, in the 400–450 millisecond time window after people receive negative emotional stimulation, the EEG signal will show higher gamma-band power and phase synchronization. They concluded that gamma-band activity is related to emotional processing [33]. In addition, in the analysis of network connections between two different emotion states in the alpha and gamma bands, we observed that there are many long-range connections among multibrain regions. According to previous studies, long-range connections are related to the functional integration of the brain under complex cognitive tasks such as emotion and behaviour control. Moreover, long-range connections mainly appear in the superior frontal gyrus, middle frontal gyrus, middle temporal gyrus, superior parietal gyrus, and middle occipital gyrus. The network connections of these brain regions contribute to emotional processing and information integration.

### 4.3. The Difference in Brain Response between Neutral and Phase Random Stimulus

The results of the difference in source response between neutral and phase random stimulus showed that, in the time period between 0.5 s and 1 s, the degree of activation of the occipital, prefrontal, and temporal lobes on the left and right sides of the neutral emotion was greater than that of phase random picture evoked emotion. Song et al. studied the difference between text and picture integration process; they found that in the early period of picture processing a larger negative N1 was evoked in the occipital region, and a negative value of N300 was evoked in the prefrontal area, which may reflect the identification process of visual stimuli and the image representation of the picture [34]. After 1.5 s of stimulation, the EEG source response intensities induced by phase random stimulation in the anterior parietal lobe of the left and right hemispheres gradually enhanced and were greater than those of neutral emotion. A comparison of the differences between brain network connections of neutral emotion and phase random picture evoked emotion showed that the differences of network connections mainly appeared in the bilateral middle temporal gyrus, right superior frontal gyrus, and left superior parietal gyrus in the delta and gamma bands. The difference between neutral pictures and phase random pictures is that neutral pictures have semantic information, and the processing of neutral pictures involves semantic information processing, while the processing of phase random pictures does not require semantic processing. Shan et al. found that the relevant brain areas for Chinese semantic processing are the left lower frontal gyrus, the left posterior inferior temporal gyrus, the joint area of the lower parietal lobe, and the superior temporal gyrus [35]. In the study by Song et al., they found that a late positive component was induced in the central, parietal, and temporal regions, which they believed might be related to the semantic activation and integration of text and pictures, respectively [34].

## 5. Conclusions

Based on SSVEP-induced emotional EEG signals, this paper uses the EEG source location method to explore the cognitive processing of the brain under emotional states from two aspects: the local brain area source response and the global brain function network connection. The results of local brain area source response show that positive and negative emotions show greater activation intensity in the temporal lobe, parietal lobe, and prefrontal lobe compared with neutral emotion. In the early stage of image processing, the higher activation intensities of the left and right occipital, prefrontal, and temporal lobe related to semantic information processing. The network connections with significant differences between positive emotion and negative emotion mainly exist in the right middle temporal gyrus and the superior frontal gyrus on both sides. The long-range connections play an important role in the process of emotional processing, especially, the connections among frontal gyrus, middle temporal gyrus, and middle occipital gyrus. In addition, both local source response and network connections in alpha and gamma frequency bands can better discriminate different emotions. The work of this paper expands the research on emotional picture processing and provides new support for the relevant researches.

## Figures and Tables

**Table 1:** MNI coordinates and Broadman partition of the centre of ROI.

ROIs	LO	RO	LP	RP	LT	RT	LMF	RMF	LSF	RSF
MNI (X)	−30	30	−28	28	−66	66	−38	38	−21	21
MNI (Y)	−78	−78	−14	−14	−52	−52	18	18	60	60
MNI (Z)	20	20	76	76	−5	−5	26	26	−7	−7
Broadman	19		6		21		46		11	
Cortical	MOG		APG		MTG		MFG		SFG	

**Table 2:** The normalized SSVEP amplitudes of positive emotion, negative emotion, and phase random picture evoked emotion (the normalized amplitudes were divided by the amplitudes of neutral emotion).

Emotion	LO	RO	LP	RP	LT	RT	LMF	RMF	LSF	RSF
Positive	0.98	1.00	0.97	1.09	1.03	1.05	1.04	1.03	1.16	1.09
Neutral	1.04	1.08	0.94	1.02	1.11	1.05	1.07	1.06	0.98	1.01
Random	0.98	0.98	0.98	1.08	0.99	0.93	0.97	0.99	0.99	0.98

**Table 3:** PLV connections with significant differences between positive emotion and neutral emotion (*P* < 0.05).

Frequency bands	Positive > neutral	Positive < neutral
Delta	RT/LT	RP/LO
Theta	—	RSF/RT; RSF/LMF
Alpha	RP/LT	LMF/RP; LSF/LMF
Beta	—	—
Gamma		RO/LO; LP/RT; RMF/RT; RMF/LP

**Table 4:** PLV connections with significant differences between negative emotion and neutral emotion.

Frequency bands	Negative > neutral	Negative < neutral
Delta	LMF/LO	—
Theta	—	LSF/LT
Alpha	LSF/RP	RMF/LT; RSF/LSF
Beta	—	—
Gamma	LSF/LO; RSF/RO	RMF/RT; LSF/RO

**Table 5:** PLV connections with significant differences between positive emotion and negative emotion.

Frequency bands	Negative > positive	Negative < positive
Delta	RMF/LMF	—
Theta	—	RMF/LO
Alpha	RP/RO; LMF/RT; LSF/LMF	RSF/RO; RSF/LP
Beta	LSF/LMF; RSF/LO	LP/LT
Gamma	RMF/LP; RSF/RO	LMF/LP

**Table 6:** There are significant PLV connections between phase random and neutral.

Frequency bands	Random > neutral	Random < neutral
Delta	RT/LO; RT/LT; LP/LO	RO/LO; RSF/RP
Theta	—	LT/LO
Alpha	—	RO/LO
Beta	RT/LT	LSF/RSF
Gamma	RT/LT	RSF/RMF; LSF/RSF

## Data Availability

The data are available upon request from the corresponding author, yingzeng@uestc.edu.cn.
